# Individuals vs. BARD: Experimental Evaluation of an Online System for Structured, Collaborative Bayesian Reasoning

**DOI:** 10.3389/fpsyg.2020.01054

**Published:** 2020-06-18

**Authors:** Kevin B. Korb, Erik P. Nyberg, Abraham Oshni Alvandi, Shreshth Thakur, Mehmet Ozmen, Yang Li, Ross Pearson, Ann E. Nicholson

**Affiliations:** ^1^Faculty of Information Technology, Monash University, Melbourne, VIC, Australia; ^2^Department of Economics, University of Melbourne, Melbourne, VIC, Australia

**Keywords:** Bayesian networks, Delphi, CREATE, BARD, reasoning, decision-making, probability, uncertainty

## Abstract

US intelligence analysts must weigh up relevant evidence to assess the probability of their conclusions, and express this reasoning clearly in written reports for decision-makers. Typically, they work alone with no special analytic tools, and sometimes succumb to common probabilistic and causal reasoning errors. So, the US government funded a major research program (CREATE) for four large academic teams to develop new structured, collaborative, software-based methods that might achieve better results. Our team's method (BARD) is the first to combine two key techniques: constructing causal Bayesian network models (BNs) to represent analyst knowledge, and small-group collaboration via the Delphi technique. BARD also incorporates compressed, high-quality online training allowing novices to use it, and checklist-inspired report templates with a rudimentary AI tool for generating text explanations from analysts' BNs. In two prior experiments, our team showed BARD's BN-building assists probabilistic reasoning when used by individuals, with a large effect (Glass' Δ 0.8) (Cruz et al., 2020), and even minimal Delphi-style interactions improve the BN structures individuals produce, with medium to very large effects (Glass' Δ 0.5–1.3) (Bolger et al., 2020). This experiment is the critical test of BARD as an integrated system and possible alternative to business-as-usual for intelligence analysis. Participants were asked to solve three probabilistic reasoning problems spread over 5 weeks, developed by our team to test both quantitative accuracy and susceptibility to tempting qualitative fallacies. Our 256 participants were randomly assigned to form 25 teams of 6–9 using BARD and 58 individuals using Google Suite and (if desired) the best pen-and-paper techniques. For each problem, BARD outperformed this control with very large to huge effects (Glass' Δ 1.4–2.2), greatly exceeding CREATE's initial target. We conclude that, for suitable problems, BARD already offers significant advantages over both business-as-usual and existing BN software. Our effect sizes also suggest BARD's BN-building and collaboration combined beneficially and cumulatively, although implementation differences decreased performances compared to Cruz et al. (2020), so interaction may have contributed. BARD has enormous potential for further development and testing of specific components and on more complex problems, and many potential applications beyond intelligence analysis.

## 1. Introduction

### 1.1. IARPA, CREATE, and BARD

Intelligence analysts are prone to the same reasoning mistakes as everyone else: groupthink, confirmation bias, overconfidence, etc. But when they produce bad assessments it can have disastrous results, such as the Weapons of Mass Destruction (WMD) reports used to justify the 2003 invasion of Iraq, which were later condemned by both sides of politics as analytically inadequate (United States Select Senate Committee on Intelligence, 2004; Silberman and Robb, 2005). So, the US intelligence community's research body, IARPA (Intelligence Advanced Research Projects Activity)^1^ has sought “structured analytic techniques” that would methodically produce better reasoned intelligence reports. Their latest, multi-million-dollar program was CREATE (CRowdsourcing Evidence, Argumentation, Thinking, and Evaluation)^2^, which specifically sought *software-based* approaches to enable *crowdsourced* structured techniques, and funded four large academic teams to pursue contrasting approaches to this end.

Our BARD team (Bayesian ARgumentation via Delphi)^3^ included computer scientists at Monash (led by Kevin Korb, Ann Nicholson, Erik Nyberg, and Ingrid Zukerman) and psychologists at UCL and Birkbeck (led by David Lagnado and Ulrike Hahn) who are experts in encoding people's knowledge of the world in maps of probabilistic causal influence: causal Bayesian Networks (BNs). A good map can provide the logical skeleton of a good intelligence report, including the probabilities of competing hypotheses, the impact of supporting evidence, relevant lines of argument, and key uncertainties. Two well-known difficulties here are eliciting sufficient analyst knowledge and amalgamating diverse opinions. So, our team also included psychologists from Strathclyde (led by Fergus Bolger, Gene Rowe, and George Wright) who are experts in the Delphi method, in which a facilitator methodically leads an anonymous group discussion toward a reasoned consensus.

The outcome of our research is the BARD system: an application and methodology whose two defining features are the construction of causal BNs and a Delphi-style collaborative process, with the aim of producing better reasoning under uncertainty and expressing it clearly in written reports. In addition, we incorporated several other features likely to improve performance, most notably: an anytime audiovisual training package, a guided incremental and iterative workflow, report templates to encourage analysts to include items often neglected, and the auto-generation of natural language text expressing some of the BN's key features. We provide a brief sketch of the system in section 3; for a more detailed picture see Nicholson et al. (2020)^4^.

### 1.2. CREATE Experiments on BARD

A key feature of IARPA's approach is the use of external testing, so their independent testing team designed a major experiment to test the effectiveness of the four CREATE approaches, including BARD. We developed, tested, and contributed some new reasoning problems that captured key elements of intelligence analysis in a simpler form, which were reviewed and included in the IARPA suite of test problems. IARPA deemed the appropriate control condition to be individuals using the Google Office Suite, since this mirrored “business as usual” for intelligence analysis. Unfortunately, IARPA's testing team relied upon retaining a large number of volunteer participants who were not significantly compensated, and attrition was so high (regardless of which of the four systems participants used) that the experiment was terminated early without obtaining any statistically useful data.

Anticipating this outcome, we designed and carried out the present study, relying on a smaller number of participants who received significant compensation. To date, it constitutes the only significant and critical experimental test of the entire BARD system used end-to-end on reasoning problems developed for CREATE. The study methodology is described in section 4, with results and discussion presented in sections 5 and 6.

Since BARD is multifaceted, and our small study is necessarily limited in the variables manipulated, it does not show how much each facet contributed to the total result. None of them are statistical confounds for this experiment, since the aim always was to test BARD as a whole. However, the contribution of each facet—and how to polish them further so they shine better together—are further research questions of great interest. In section 2, we briefly review the most relevant theory and previous experimental results, including two experiments our team performed to separate BARD's BN construction from its Delphi collaboration. This review supports the view that each of BARD's facets most likely contributes *positively and cumulatively* to total BARD performance. We hope that future research will improve, validate, and measure each contribution.

## 2. Background

### 2.1. Intelligence Analysis Problems

Intelligence analysis typically requires assessing the probability of some conclusion based on available pieces of evidence, and writing reports for decision-makers to explain that assessment. To express those probabilities, US analysts are expected to use a standard verbal terminology corresponding to defined numerical ranges (e.g., “very likely” means 80–95%) as specified in ICD-203, which “establishes the Intelligence Community (IC) Analytic Standards that govern the production and evaluation of analytic products” [Office of the Director of National Intelligence (ODNI), 2015]. The same conclusions are often reassessed periodically as new evidence arises. This sort of intelligence analysis requires a type of reasoning under uncertainty that is not unusual: similar reasoning is required in many other domains, and we hope that BARD's success with our test problems will ultimately be transferable to many real-world problems.

To test the BARD system, our team needed to develop new reasoning problems that captured the key elements of intelligence analysis in a simpler form. Basic scenarios and evidence are presented in written form, and answers must ultimately be given in written form, but participants can use other means (e.g., BNs, pen-and-paper calculations) in between. In each of our short reasoning problems, we incorporated a major reasoning difficulty likely to lead to some qualitatively incorrect conclusions and explanations, and we also tested the accuracy of quantitative estimates. Our reasoning problems were developed and tested by our London-based cognitive psychologists.

We used two of these problems in this experiment. The Kernel Error problem involves the cognitive difficulty known as “explaining away.” For example, if my wet lawn must be caused by either a sprinkler or rain (or both), and these two causes are each sufficient and otherwise independent, then seeing the wet lawn raises the probability of both possible causes. However, if I discover that it rained, this entirely “explains away” the wet lawn, and the probability of the sprinkler should be lowered to its initial value. Our team's psychology experiments with Kernel Error formally confirmed what computer scientists have informally observed: people have difficulty readjusting their probabilities appropriately (Liefgreen et al., 2018).

The Cyberattack problem involves the cognitive difficulty known as dependent evidence. For example, how much additional weight should we give to a second medical test result if we know that the second test was of the same type as the first? This depends on how the results are correlated, e.g., how often errors in the first test will be caused by factors that will also cause errors in the second test. Even if people have precise figures for this, our team's psychology experiments with Cyberattack formally confirmed that people find it difficult to combine dependent evidence accurately (Pilditch et al., 2018).

### 2.2. Probabilistic and Causal Reasoning Errors

Psychological research has revealed many difficulties people have with both probabilistic and causal reasoning (Kahneman et al., 1982; Hahn and Harris, 2014; Newell et al., 2015). To summarize a very large literature:

- One general factor that increases the probability of such errors is simply complexity. Facing a mass of interconnecting evidence and long lines of argument, it is easier to make an error somewhere along the line in assessing the impact of evidence on a conclusion.
- Another general factor is specific dependence patterns that people find surprisingly difficult. Besides explaining away and dependent evidence, these include “screening off,” i.e., when knowledge of the state of a common cause renders two dependent effects independent of each other, and mistaking correlation for direct causation when a hidden common cause is far more likely (Gopnik et al., 2001; Lagnado and Sloman, 2004; Kushnir et al., 2010; Pearl and Mackenzie, 2018).
- A third general factor is the common biases in the way people express and update their probabilities, such as overconfidence, i.e., exaggerating the probability of likely events and the improbability of unlikely events (Moore and Healy, 2008); conservative updating, i.e., inadequately weighting new evidence when revising beliefs (Kahneman et al., 1982; Matsumori et al., 2018); base-rate neglect, i.e., inadequately weighting the priors (Welsh and Navarro, 2012); and anchoring, i.e., depending too much on an initial piece of information (the anchor) (Kahneman et al., 1982).

### 2.3. BNs to Reduce Reasoning Errors

A key reason for IARPA's interest in structured representations is to reduce such cognitive difficulties when analyzing problems (Heuer, 1999). Causal BNs are particularly well-suited for the task, since they explicitly represent and accurately combine both probabilistic and causal information.

Formally, a BN is a directed, acyclic graph whose nodes represent random variables, and whose arrows represent direct probabilistic dependencies, often quantified by conditional probability tables (CPTs) associated with each node. In causal BNs, each of these arrows also represents direct causal influence—hence, they can also predict the effects of decisions to intervene. Users can enter exact or uncertain evidence about any variables, which is then efficiently propagated, updating the probability distributions for all variables. Thus, causal BNs can support and perform predictive, diagnostic (retrodictive), explanatory, and decision-oriented probabilistic reasoning. For more technical details, see Pearl (1998), Spirtes et al. (2000), and Korb and Nicholson (2011).

But how does constructing a BN help people avoid reasoning errors, rather than merely reproducing them? Reasoning errors aren't bad beliefs; they are bad ways to develop or combine beliefs. So, BN assistance doesn't depend on all the analysts' beliefs being true, it just enables analysts to accurately draw the conclusions that are implied by their own beliefs. It's analogous to using a calculator to help avoid arithmetical errors: provided that people enter the numbers and operations they believe are correct, the calculator can be relied upon to combine them accurately. In constructing BNs, analysts must explicitly think about and identify the causal structure (rather than make implicit assumptions about it). The model then requires all the relevant probabilities to be entered (so none of these can be neglected). The BN calculations then automatically avoid almost all the errors discussed above. The way modeling with BNs helps avoid errors has been explained and/or empirically verified for multiple specific reasoning difficulties: base-rate neglect (Korb and Nyberg, 2016); confusion of the inverse, i.e., interpreting the likelihood as a posterior (Villejoubert and Mandel, 2002); the conjunction fallacy, i.e., assigning a lower probability to a more general outcome than to one of the specific outcomes it includes (Jarvstad and Hahn, 2011); the jury observation fallacy, i.e., automatically losing confidence in a “not guilty” verdict when a previous similar conviction by the defendant is revealed (Fenton and Neil, 2000); and most recently, the zero-sum fallacy, i.e., not recognizing when a piece of evidence increases the probability of *both* a hypothesis and its most salient rival (Pilditch et al., 2019). Exceptions to this rule might be reasoning errors that arise from mistaken ways to express individual beliefs, e.g., ambiguities in variable definitions, or overconfidence in the initial probabilities assigned. For such issues, the critical discussion engendered by structured social processes may be more useful, per sections 2.4 and 2.5.

More generally, given their ability to embody normatively correct reasoning, causal BNs have been used to analyze common fallacies in informal logic (Korb, 2004), analyze and assess a variety of arguments in criminal law—where they have exposed some common errors in evidential reasoning (e.g., Fenton et al., 2013; Lagnado et al., 2013), analyze human difficulties with reasoning under uncertainty (e.g., Hahn and Oaksford, 2006; Hahn, 2014), model human knowledge acquisition while solving complex problems (e.g., Holt and Osman, 2017), and as a proposed method for argument analysis (Korb and Nyberg, 2016). In practical contexts, they have been deployed to support human reasoning and decision making under uncertainty in such diverse domains as medicine (e.g., Flores et al., 2011; Sesen et al., 2013), education (e.g., Stacey et al., 2003), engineering (e.g., Choi et al., 2007; Bayraktar and Hastak, 2009; Misirli and Bener, 2014), surveillance (e.g., Mascaro et al., 2014), the law (e.g., Fenton et al., 2013; Lagnado and Gerstenberg, 2017), and the environment (e.g., Chee et al., 2016; Ropero et al., 2018).

Many BN software tools have been developed to assist in building, editing, evaluating, and deploying BNs. These include Hugin^5^, GeNie^6^, Netica,^7^ AgenaRisk^8^, BayesiaLab^9^, and a plethora of research software tools, e.g., Elvira^10^, R BN libraries^11^, BNT^12^, SamIam^13^, and BayesPy^14^. However, all of these tools assume that the user understands BN technology (or they offer only rudimentary help), and they assume the user knows how to translate their knowledge of a causal process or argument into a Bayesian network. In the BARD system, we improved on this first generation of BN tools by providing far better training and guidance (see section 3.2), and by providing a structured workflow that draws on new BN “knowledge engineering” concepts and best practices (see section 3.3).

### 2.4. Delphi Groups to Improve Reasoning

There is considerable evidence that decision making by groups, either by reaching consensus or by amalgamation, can produce better outcomes than decision making by individuals (e.g., Salerno et al., 2017; Kugler et al., 2012; Charness and Sutter, 2012; Straus et al., 2011). However, there are also well-known problems that arise with group interactions, e.g., anchoring, groupthink, and psycho-social influences (for more details, see Kahneman et al., 1982; Mumford et al., 2006; Packer, 2009; Stettinger et al., 2015). Groups also have potential logistical advantages in that subtasks can be divided among members and/or performed by the most competent.

A number of methods have been developed over the years that attempt to harness the positives of groups while pre-empting or ameliorating the negatives. One of the best-known is the Delphi technique (e.g., Linstone and Turoff, 1975), an example of a “nominal group” technique: the group members never actually meet, but rather, interact “remotely.” The defining characteristics of a Delphi process (e.g., Rowe et al., 1991) are: *anonymity* to reduce the influence that powerful or dogmatic individuals can have on group judgments; *iteration* with *feedback*, which allows participants the chance to reconsider and improve their responses in the light of information from other group members; and *aggregation* (or collation, if responses are qualitative in nature) of group responses, often done by a *facilitator*—who can also assist by reducing unproductive exchanges and encouraging task completion (but avoids making original contributions). At least for short-term forecasting problems and tasks involving judgements of quantities, Delphi has generally shown improved performance compared to freely interacting groups or a statistically aggregated response based on the first-round responses of individual participants (Rowe et al., 1991).

### 2.5. Delphi for Constructing BNs

Recently, one study used a form of Delphi for point-estimate CPT elicitation (Etminani et al., 2013), while for BN structure elicitation Serwylo (2015) pioneered online crowdsourcing and automated aggregation (albeit non-Delphi). Some of the present authors proposed a Delphi-style elicitation of BN structure in an epidemiological case study (Nicholson et al., 2016). However, BARD is the first system to use Delphi for developing and exploring an entire BN model, including variables, structure and parameters, and also for more complex reasoning problems.

The major difficulty in using Delphi here is that both the workflow and the output are complex: the workflow necessarily involves multiple, logically dependent steps, and users should be encouraged to improve their complex answers iteratively by repeating steps. One approach would be for each participant to complete the entire process before discussing their work with others, but this means they would learn nothing from others during the process and have complicated outputs to assess and discuss at the end. Another approach would be to use a traditional Delphi process at each step and make the workflow strictly linear, but this loses all the advantages of iterative development, and requires synchronized participation. BARD resolved this dilemma by using a compromise: “Real-Time Delphi” (see section 3.3). One crucial achievement of this experiment is to demonstrate the feasibility of combining Delphi with BN construction in this way.

In real-world applications, the relevant probabilities may come from either data, such as available studies on the false positive and false negative rates for a medical test, or expert opinion, such as the relative risks of new medical treatments where there is little data available. In either case, there may be disagreement and uncertainty. Instead of a single point probability, the available information is then better summarized as some sort of probability distribution or interval, which may be interpreted as meta-uncertainty about the appropriate point probability, and called a “vague” probability. There have been various protocols proposed for eliciting and combining such probabilities from multiple experts, such as 3-point methods (e.g., Malcolm et al., 1959; Soll and Klayman, 2004), a 4-point method (Speirs-Bridge et al., 2010), and the IDEA protocol (Hemming et al., 2018a); however, these have not been integrated into any of the commercial or research BN software tools. Instead, these protocols are applied externally to the BN software and then incorporated by the BN model builder (e.g., Nicholson et al., 2011; van der Gaag et al., 2012; Pollino et al., 2007; Hemming et al., 2018b). Uniquely, BARD integrates elicitation tools of this kind with BN construction. However, the test problems in this experiment specify appropriate point probabilities in the problem statements, in order to simplify the task and yield uncontroversial, normatively correct solutions. So, assessing the effectiveness of BARD for vague probabilities must await future research.

### 2.6. Checklists for Improving Reasoning

One of the simplest structured techniques is the checklist—yet, it has proven highly effective in reducing errors in such challenging expert tasks as piloting aircraft and, more recently, in performing medical surgery (Russ et al., 2013). Effective checklists are carefully designed to provide timely and concise reminders of those important items that are most often forgotten. For CREATE, IARPA had already identified important general elements of good reasoning that are frequently omitted, e.g., articulating competing hypotheses, and noting key assumptions (Intelligence Advanced Research Projects Activity, 2016). This suggested that something like a reasoning checklist could be useful, if added to the BARD system.

For BN-building, the functions of a checklist are implicitly fulfilled by our stepwise workflow with step-specific tips, and associated automated reminders. For report-writing, we implemented the checklist idea more explicitly in the form of a report template with section-specific tips, and associated automatic text generation (see section 3.4).

### 2.7. Experiments Separating BN Construction From Delphi Collaboration

The BARD team performed two other critical experiments on the BARD system, reported in detail in the references below, which provide some evidence that its two principal features—BN construction and Delphi groups—both contribute positively and cumulatively to BARD's total performance.

In the SoloBARD experiment (Cruz et al., 2020), individual participants used a version of BARD without any social interaction to solve three of the reasoning problems our team developed, including the Kernel Error and Cyberattack problems used in this experiment. The control condition consisted of individuals provided only with Microsoft Word and IARPA's generic critical thinking advice. The results showed much better performance from the individuals using SoloBARD. This provides some evidence for the feasibility and effectiveness of BN construction (supported by BARD's other non-social features, such as templates) to analyze probabilistic reasoning problems and produce written reports.

In the Structure Delphi experiment (Bolger et al., 2020), individual participants who had previously used BARD were asked to analyze some of our other reasoning problems they had not previously seen, but only for the critical and most distinctive subtask in BN construction: selecting the right variables and causal structure. All other subtasks in solving our reasoning problems are similar to tasks for which Delphi has already been shown to be effective in prior literature. Individual participants were shown the structures purportedly proposed by other members of their Delphi group (although, in fact, generated earlier by similar participants and curated prior to the experiment) and invited to rate these structures and revise their own. The results showed they made substantial improvements over their initial responses, both in the top-rated structures and in the revised structures. This provides some evidence that BARD's Real-Time Delphi social process is an additional positive contributor to performance in analyzing probabilistic problems.

We did not perform any experiment directly comparing groups using BARD to groups using Google Docs. This was partly due to our resource limitations, and also to its lower prioritization by IARPA. Given our combined experimental results, we think it highly unlikely that groups with Google Docs could have outperformed groups with BARD on these particular probabilistic reasoning problems. Nevertheless, sorting out the exact independent and combined contributions of BARD's BN-building and structured social processes vs. unaided, unstructured group processes remains an interesting research task for the future.

## 3. The BARD Application

### 3.1. Overview

BARD (Nicholson et al., 2020) supports the collaborative construction and validation of BN-based analyses in a web application, in a Delphi-style workflow. Analysts in small groups, optionally assisted by a facilitator, are guided through a structured Delphi-like elicitation protocol to consider and represent their relevant knowledge in a causal BN augmented by descriptive annotations. BARD provides tools to assist the elicitation of a causal BN structure and its parameters, review and build consensus within the group and explore the BN's reasoning in specific scenarios. BARD encourages analysts to incrementally and iteratively build their individual BNs and seek regular feedback through communication with other group members and the facilitator. The group may decide to adopt the highest-rated individual BN or a facilitator can assist in the production of a consensus model. From an individual or group BN, BARD auto-generates an outline of a structured verbal report explaining the analysis and identifying key factors (including the diagnosticity of evidence and critical uncertainties). Analysts and the facilitator can revise this into an intuitive narrative explanation of the solution, using a structured template prompting users to incorporate elements of good reasoning.

### 3.2. Better Training for Building BNs

Our experience is that substantial training is required to model effectively with BNs. For example, the standard BayesiaLab training is conducted in a 3-day course^15^, while Bayesian Intelligence Pty Ltd offers 2-day training as standard^16^. However, the requirements of testing and evaluation in the IARPA CREATE program limited upfront training to 4 h of online, individual, self-paced training, without any input or assistance from a human instructor.

BARD upfront training developed for CREATE is delivered as condensed but high-quality audiovisual e-courses, with corresponding practical exercises, example solutions, and context-sensitive help and tips embedded in the software. They cover the fundamentals of Bayesian network modeling, how teams function in BARD, the differences in the responsibilities of facilitators and analysts, and details on how to use the BARD software itself^17^.

### 3.3. Better Workflow for Building BNs

**Figure 1:**
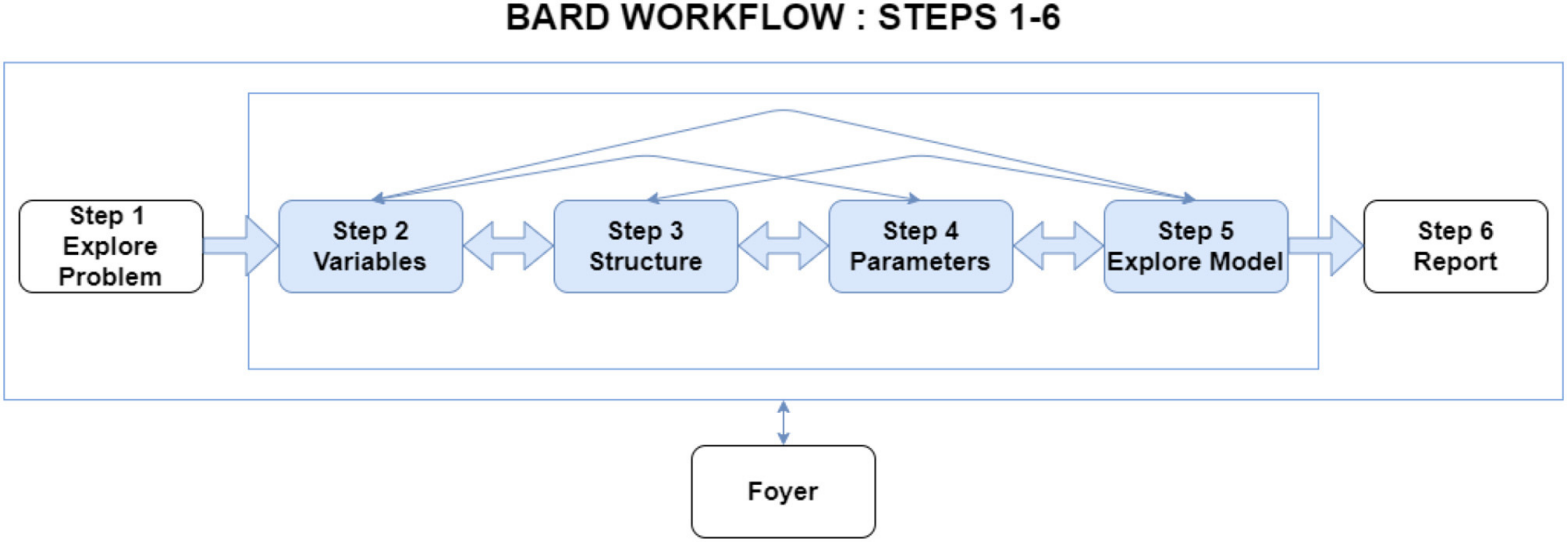
BARD's workflow consists of six steps. Analysts and the facilitator can move flexibly backwards and forwards between steps.

The BARD workflow decomposes the task into a logical series of six smaller steps (see Figure 1). Step 1 focuses on understanding the problem for analysis, particularly identifying hypotheses as well as the most relevant factors and evidence. In Steps 2 to 5, participants build a BN model of the analytic problem, broken down into variable selection (Step 2), adding arrows to define the structure (Step 3), parameterizing the model to specify the probabilities (Step 4), and then exploring and validating the BN's reasoning on specific scenarios (Step 5). Finally, the participants individually and collectively construct a written report (Step 6).

**Figure 2:**
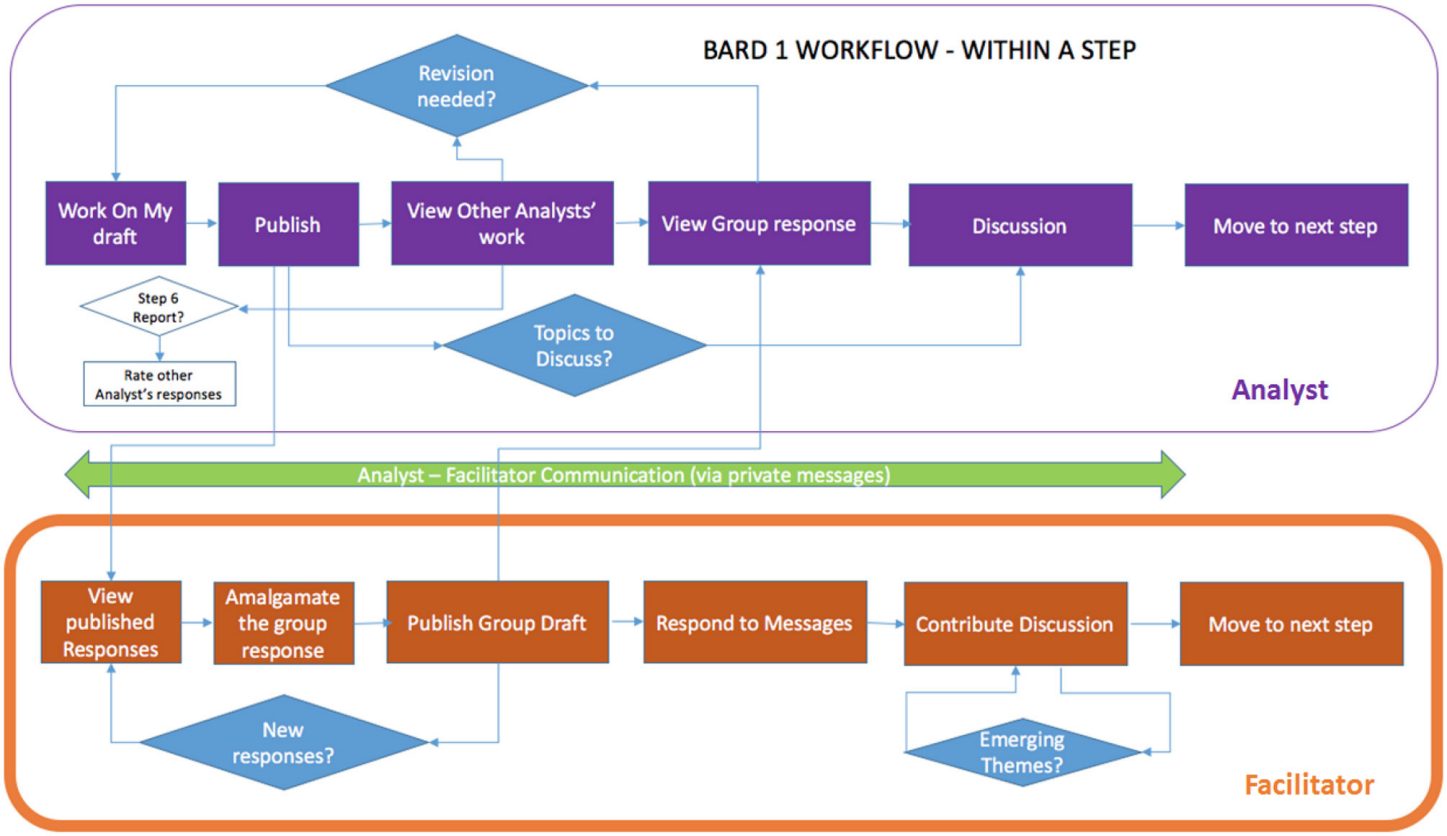
High level representation of the BARD workflow within a step for analysts and the facilitator.

At each of the steps, analysts are required to first work on their solution in isolation (blinded to other responses) and then “Publish” their work (which makes it available to other analysts), before they can view other analysts' work or the current group solution (produced by the facilitator), and discuss them via the step-specific discussion forum (see Figure 2). Publishing also allows analysts to move forward to the next step. When present, a facilitator's role is to: support the team's progression through the steps in terms of timeliness and focus; optionally synthesize the team's work in the “group” solution (both BN and report) with minimal original contributions of their own; encourage review, feedback, and discussion; and submit the final analytic report.

Thus, apart from the initial response requirement, at each step group members are free to progress to subsequent steps at their own pace and can move flexibly backwards and forwards between steps. This BARD workflow is based on a “roundless” Delphi variant called “Real-Time Delphi” (Gordon and Pease, 2006), where the sub-steps of providing individual responses, viewing information from other participants, and improving responses are not controlled by the facilitator, but rather, where the transitions occur immediately, i.e., in “real time.” This allows far more flexibility about when the participants can make their contributions and speeds up the Delphi process, since analysts do not have to wait for the facilitator to amalgamate or collate responses, as well as reducing the need for facilitation. It also allows users to return to earlier steps to expand on their answers, since BNs are best built iteratively and incrementally (Laskey and Mahoney, 1997, 2000; Boneh, 2010; Korb and Nyberg, 2016). The trade-off is that, since the participants can see each other's responses directly, rather than after amalgamation or collation, some of the biases deriving from direct interaction that Delphi is designed to eliminate may re-emerge.

At Step 6, analysts can also rate their own and other analysts' reports on a 10-point scale; after rating a report, they can see their own rating and the current average rating. This feature was introduced as a quantitative high-level assessment to help focus discussion, as well as providing guidance to the facilitator on which report(s) to use as the basis for the team solution. However, in the absence of a facilitator, these ratings can also be used as input to an algorithm to automatically select an individual report as the team solution (see section 4.3.2).

Using this workflow, a team can methodically produce an analytic report explaining the members' collective answer to the problem and their reasoning behind it.

### 3.4. Report Templates and Automated BN Explanations

BARD pre-populates the written report workspace with a few generic headings, along with explanatory tips for each heading. These function as checklist-style reminders and placeholders for these general elements, e.g., the relevant hypotheses and their prior probabilities, and they also clarify the presentation for the reader. Participants are encouraged to include tables or figures, such as an image of the BN structure, if these enhance clarity further. We note that TRACE, one of the other four CREATE projects, also experimented successfully with flexible report templates (Stromer-Galley et al., 2018), which supports the view that they make some positive contribution.

In conjunction, we developed a rudimentary AI tool for generating text explanations of the relevant BN features, and organized this text under the same template headings so that it could readily be copied or imitated in the written reports. The reason for providing such assistance is that, especially when BNs become more complex, it can be difficult to understand the interaction between evidence items and their ultimate impacts on the conclusion. Although the BN will have calculated this accurately, well-reasoned reports demand that the impact be explained verbally, and it helps if the BN can explain itself^18^.

## 4. Methodology

This study was approved by the Monash University Human Research Ethics Committee, with the plan^19^ lodged with the Open Science Framework (OSF)^20^ and approved by them and by the IARPA CREATE program.

### 4.1. Participants

**Power Analyses:** We conducted separate power analyses for our *t*-tests and repeated measures ANOVAs, both assessed for a statistical power of 0.8. We chose large effect sizes to reflect that only substantial improvements over the control would be sufficient to justify adopting the BARD system. For the *t*-test of the difference between two independent means at the 5% level of significance (one-sided), with equal sample sizes and a very large effect size (Cohen's *d* = 1.33, equivalent to a 20% improvement of BARD over a control mean score of μ_*k*_ = 20 with equal σ = 3 across conditions), we calculated an indicative sample size of 16 score observations split evenly between BARD and the control. Assuming 8 individuals were recruited into each BARD team to form a single test score, this would require 72 individuals in total. Assuming a larger standard deviation (σ = 5), a large effect size (*d* = 0.8) and retaining other assumptions, we calculated 21 observations per condition requiring 189 individuals in total. For a repeated measures within-factors ANOVA at the 5% level of significance (one-sided), with equal sample sizes, across two periods, with two factors and a very large effect size (partial η^2^ = 0.735, equivalent to Cohen's *d* = 1.33), we calculated an indicative sample size of 4 observations per condition requiring 36 individuals in total. For a large effect size (partial η^2^ = 0.39, equivalent to Cohen's *d* = 0.8) we calculated 8 observations per condition or 72 individuals in total. To cater for the worst case among these analyses, we set a minimum recruitment target of 189 individuals.

**Recruitment Methods:** We recruited participants via social media (using Monash University's Facebook, LinkedIn, and Twitter pages to advertise for volunteers), Monash University student organizations, and the Monash Psychology department's SONA system. All participation was voluntary, and participants could withdraw at any time prior to completion of the study while retaining any compensation earned. All responses were fully anonymized (including all IP address information). A short quiz^21^ including probabilistic reasoning questions and personality questions was completed by all participants when registering for the experiment. This was not used to select applicants for the experiment or assign their condition, but was used later to help allocate the facilitator role within BARD teams.

**Sample Size, Conditions, and Demographics:** We attempted to over-recruit since the rate of attrition could not be known in advance, and we succeeded in obtaining 295 registrations. These potential participants were 18–57 years old with a mean of 29.7 and a standard deviation of 7.2 years. By optional self-identification, there were 139 females, 141 males, and 15 others. The target population was English-speaking adults with some undergraduate experience, so individuals who had not yet completed high school education or were younger than 18 were excluded.

Following the randomized control trial (RCT) standard, these potential participants were selected randomly into two conditions for between-condition comparisons. After asking them to confirm their availability for the respective time commitments required, we began the experiment with 256 participants. 58 control (K) participants were asked to work individually at any time to produce reports, using the Google Suite tools and (if desired) some pen-and-paper techniques (see section 4.3.3). The remaining 198 experimental (X) participants were asked to work collaboratively and synchronously in teams of 6–9 using the BARD tool. By self-identification, K contained 20 females, 37 males, and 1 other, while X contained 98 females, 86 males, and 14 others.

Participants were kept blind of their condition in the sense that they were not informed about the nature of any other conditions. However, blinding was necessarily imperfect, in that many participants would have heard of the IARPA CREATE program and/or BARD independently of the experiment itself, and may have been aware that the BARD project utilizes BN technology. In particular, some K participants may have been aware that they were not using the technology under development and performing as controls. Of course, every participant was trained explicitly only in the tools actually required for their condition.

**Compensation:** Participants were compensated for adequate participation in each session in the form of a GiftPay^22^ voucher, and those who participated in all sessions received a bonus. All participants (X and K) were required to complete the upfront training to receive compensation. In each of the 5 problem-solving weeks, X participants were required to attend joint problem solving sessions and actively work on their reports to receive compensation, while K participants were only required to complete their report. For the optional webinars (see section 4.3), attendance was sufficient.

### 4.2. Materials

Three analytic problems were selected for the study; all were probabilistic in nature and ideally suited to being solved using Bayesian networks: (A) Smoking and Cancer^23^; (B) Kernel Error (Liefgreen et al., 2018); and (C) Cyberattack (Pilditch et al., 2018).

All problems had corresponding marking rubrics, with those for B and C developed previously by our team's cognitive psychologists, and a similar format used here for A^24^. Participants were explicitly asked to provide some specified probabilities, but also asked to justify those answers. In the rubrics, assessors were provided with both the correct answers and a short list of specific observations which ought to feature in any sound and thorough justification, e.g., that one evidence source is more reliable than another. Assessors awarded one point for each answer and each observation that participants fully included, and a half point for each observation that was only partially included. The final rubric score was simply the sum of these points.

The nature of these rubrics entails that there are a large number of available points (13, 38, and 34 respectively), but the mean proportion of these points obtained by participants tends to be low. It is less clear to participants which items to include in their justifications than in their answers, and in ordinary life people frequently give shorter, partial justifications that leave some relevant facts unstated. Hence, even participants with correct answers obtained by correct reasoning are likely to omit some point-scoring observations from their justifications. Conversely, even participants who give incorrect answers or use incorrect reasoning are likely to score some points in their justifications. To avoid this “random noise” inherent to scoring justifications, the SoloBARD experiment (Cruz et al., 2020) also compared points scored only from the answers to the explicit questions, and found a much greater effect size (Glass' Δ = 1.4) in favor of SoloBARD—but we did not propose or perform this analysis for our BARD experiment.

Unlike the SoloBARD experiment, we split Problems B and C into two parts. Part 1 introduced a new scenario with relevant evidence and questions that needed to be answered. Part 2 of each problem was presented in the following week, building on the first by adding new evidence to the problem descriptions and then asking additional questions about its impact. BN models readily allow for such “phased” problems, and BARD takes advantage of that in allowing “scenarios” to be built incrementally along with the models used to analyze them. So, both K individuals and X teams were able to build on their analyses for Part 1, even though those questions were not repeated and their rubric scores did not carry over to Part 2.

In Part 2 of both problems, participants must cope with more variables and more dependencies between them—which makes the problems computationally more difficult than in Part 1. Furthermore, these additional elements introduce the major cognitive difficulties designed into these problems. For both reasons, the Part 2 questions should be more difficult for participants, and we expected them to achieve a lower proportion of the available marks. Furthermore, we expected the advantage of using BARD to become more pronounced. To test this secondary hypothesis, we used a separate ANOVA for each of these problems to detect any significant interaction, despite the small loss of statistical power in detecting the main effect.

In the SoloBARD experiment, Problems B and C (not divided into parts) seemed to present roughly the same difficulty for participants: controls obtained roughly the same proportion of the available points in both problems, and so did participants using SoloBARD. Our Problem A was structurally comparable to the first part of the other two problems, and hence not particularly difficult nor divided into parts. It is similar to example BN problems common in introductory undergraduate Artificial Intelligence courses, and partly intended to provide additional training for both X and K in conjunction with the associated webinar on how it can be accurately solved, before they proceeded to the more difficult problems.

The problem-solving was conducted over 5 consecutive weeks, with the webinars, training, and problems being presented in the sequence shown in Table 1.

**Table 1:** Experiment schedule by week: for webinars, training, and problem-solving.

**Week**	**Webinar**	**Task**
0	Welcome	Training
1	Q&A on Training	(A) Smoking and Cancer
2	Solution and Q&A for (A)	(B) Kernel Error, Part 1
3	–	(B) Kernel Error, Part 2
4	Solution and Q&A for (B)	(C) Cyberattack, Part 1
5	–	(C) Cyberattack, Part 2

### 4.3. Design and Procedure

#### 4.3.1. The Variables

The variable under manipulation was the tool and associated training used for analyzing problems and writing solutions; the dependent variable assessed was performance in producing these solutions. X and K membership was assigned uniformly randomly, using random.org to select a sufficient number of participants for K. This implicitly controlled for other independent variables; those measured, via the registration quiz and BARD's usage monitoring, were: Education level (high school, some college, BA, MS, PhD); Probability/Stats education; Sex; Nationality; Age (≥18); Total login time.

Very high attrition rates were observed in all preliminary studies by CREATE teams, including pilot studies for this experiment in both X and K: up to 50% per week, which would have been unsustainable over the course of the experiment. We made several adjustments to minimize and cater for attrition, most notably by encouraging frequent social engagement. X team members were required to work synchronously; and for both X and K we introduced “webinars” (i.e., online seminars) presented by a member of the experimental team that provided additional training and Q&A; these were voluntary, but (apart from the initial Welcome) participants received additional compensation for attendance.

#### 4.3.2. Experimental Condition (X)

**Training:** For this study, compulsory upfront X training consisted of only 2 h of the BARD e-courses for analysts, delivered individually using a Learning Management System (Moodle). Participants were then asked if they were willing to take on the facilitator role. Those who answered “yes” and completed the short, optional facilitator e-course were subsequently considered as prospective facilitators. All e-courses remained accessible via the BARD platform throughout the experiment.

The four different webinars were held according to the week-by-week schedule in Table 1, and within each week, the scheduled X webinar was presented four times on weekday evenings to cater for participant availability and keep the numbers in each session manageable. Their respective aims were: to welcome and introduce participants to the experiment and encourage them to do the training; to answer any questions that arose from the training; to review the BARD gold-standard solution for Problem A and answer any questions; and to conduct a similar review for Problem B. These “gold-standard solutions"” were simply plausible example solutions we constructed, including the associated BNs, that would have achieved the maximum possible rubric score. PowerPoint slides and BARD walkthroughs were used to explain these solutions and how to use BARD to develop them^25^. No webinar was conducted after Problem C, as there were no subsequent problems where participant performance could benefit from further retention or training; however, participants were sent the gold-standard solution via email.

**Assignment to teams and roles:** X participants were permanently assigned to one of six timeslots spread over three weekday evenings, consistent with their stated availability and our capacity, and asked to keep this timeslot free for participation throughout the experiment. They were then randomly assigned to BARD teams within this timeslot before each problem cycle. Reassignment was another modification to cope with attrition, by maintaining participant numbers within each team. Initially, there were 25 teams made up from 198 people selected for the X condition, but attrition reduced the number of teams across the experiment (see section 5.1). Teams were assigned 6–9 members (except for one team of 5 during the final problem) with an average of 7.3 members for the experiment. We expected some attrition within teams during each problem, i.e., that not all assigned members would actively participate, so the numbers assigned were slightly generous.

As described in section 3, each BARD team had one facilitator and the remainder were analysts. The prospective facilitators were assigned to teams first and distributed as evenly as possible, since BARD includes functionality for facilitators to be replaced. Within each team, the participant with the highest score on the quiz done at registration was selected as the facilitator.

**Workflow:** BARD's workflow was designed to allow asynchronous problem solving, i.e., with no real-time communication. However, to increase social engagement, team members in this experiment were required to work synchronously online during their allocated 2-h sessions, which was feasible because almost all participants were within the local AEST timezone. While lab-based experimentation would have been even better for combating attrition, as used in Cruz et al. (2020), here our resources were insufficient. Once a problem was “opened” at the start of the team's scheduled session, the participants still had access to BARD and the problem for the remainder of the week until it was “closed” at midnight on Sundays, and so could continue to work on it after the scheduled session time, albeit without additional compensation. In practice, while some participants continued to work on the solution the same night, no participants came back on subsequent days.

**Report submission:** When the problem was closed, the rules for report submission were:

1. If the facilitator has already submitted a final report, that report will be assessed. The facilitator was trained and instructed to produce the report by either:
  - incorporating elements from any or all of the individual reports, or
  - choosing what appears to be the best analyst report, based on team consensus via the discussion forum and/or ratings^26^.
2. If the facilitator does not submit a report, then among those reports given a rating by at least two analysts, BARD auto-submits the one with the highest mean rating.
3. If there is no report rated by at least two analysts, then BARD auto-submits the longest non-blank analyst report^27^.

#### 4.3.3. Control Condition (K)

**Training:** K individuals received webinars and upfront training for their own tools that were as similar to X as practical^28^. Nevertheless, the content between the X and K webinars differed significantly. K used Google Suite, and their upfront training consisted of an e-course developed by IARPA called the “Guide to Good Reasoning”^29^, which provided generic training on how to reason and solve problems, including avoiding the common analytic errors IARPA had already identified.

Webinars followed the same week-by week schedule described in Table 1 and had similar aims. Each webinar was presented three times within each week, and individuals nominated the timeslot they preferred at the beginning of the experiment. In the webinars following Problems A and B, we presented versions of the gold-standard solutions with almost identical text to those for X, but stripped of any allusion to the BARD tool. We used PowerPoint slides^30^ to introduce and explain how “frequency formats” and “chain event graphs” could be used to accurately calculate the answers (see Gigerenzer and Hoffrage, 1995), and also how the elementary probability calculus could be used as a supplement or alternative method, albeit more mathematical and less intuitive. These are the best available pen-and-paper techniques for probability calculation, and were sufficient, in principle, for solving all our problems precisely.

The main motivation for presenting these techniques was to encourage continued participation. As discussed in section 2, the more “ecologically valid” and favorable comparator would have been individual analysts working on problems without any special training in probability calculation, as used in Cruz et al. (2020). For intelligence analysis, these pen-and-paper techniques aren't part of business as usual, and moreover, are not a viable alternative to BNs: although a feasible low-tech alternative for these simplified test problems that require computing a few explicit and precise probabilities, they rapidly become too unwieldy and difficult as problems become more complex or vague. Nevertheless, although we expected this training to improve the performance of K, we reasoned that if X could still outperform K here, then it would outperform an untrained K by at least as great a margin.

**Workflow:** For ecological validity, K participants worked on each problem individually. A welcome side effect was that it allowed us to maintain the study's statistical power despite limited funding for participant compensation. Analysts in K were provided with individual Google Drive folders containing the Good Reasoning Guide, and for each week the relevant problem statement and blank “Answer Document.” K had 58 participants initially, with 51 completing training, and further attrition leading to smaller numbers for problem-solving (see section 5.1).

**Report submission:** For K, problems were “opened” on Monday simply by releasing the problem description, and participants had the entire week to work on their report at their convenience. They were free to enter their solution in the Google Drive anytime between the opening and the close on Sunday at midnight, and any non-blank Answer Document was assessed.

#### 4.3.4. Marking

Six markers were engaged with proven marking ability: fluency in English and a background in academic marking. Marker training included a review and discussion of an Assessment Guide^31^, as well as a joint session marking example reports. Markers were trained to adhere as closely as possible to a literal interpretation of the problem rubrics and ignore redundant information. Markers were obliged to work independently of each other and BARD project members. Reports were anonymized and marking done blind; in particular, markers were not informed whether they were marking an X or K report.

Markers could not be kept completely blind, however, since only the BARD reports were generated using a structured template, with encouragement to include BARD graphics. As discussed in section 3.4, these are beneficial features of BARD, both because they remind users to provide some oft-neglected content and because they help to present that content more clearly. The potential problem here is not that markers might give legitimate rubric points for providing such content, but rather, that they might become biased in their interpretation of which reports are providing it, and hence illegitimately award points to X or not award points to K. Fortunately, the items awarded rubric points are all very specific pieces of information and it is difficult to misinterpret whether these are provided. However, we endeavored to minimize any such bias by explicitly urging markers to avoid it, and informing them that their performance would be tested for it: some fully anonymized K reports would be camouflaged to appear as X reports and vice versa.

### 4.4. Statistical Design

The design was pre-registered with The Open Science Framework (OSF)^32^, and in accordance with our IARPA contract, stated that inferences about our main hypothesis would be primarily based on 80% and 95% confidence intervals (CIs) for condition means, and standardized effect sizes. We proposed to show that X had the higher mean rubric scores overall (across all three problems), with favorable non-overlapping CIs taken as confirmation of the hypothesis. We also present below the results of some more usual null hypothesis significance tests.

We did not explicitly set a precise target effect size. CREATE, however, had specified at the outset its own performance goals for “Quality of Reasoning” to be achieved by the end of each of its three Phases: Cohen's *d* (pooled) of at least 0.25 (small), 0.5 (medium), and 1.0 (large) respectively. *d* ≥ 1.0 was an ambitious final target, since for structured analytic techniques, this is a major effect that has rarely been robustly achieved. For example, “Argument Mapping” (AM) is a well-known software-supported structured technique where an analyst makes a non-causal, non-parameterized tree diagram to illustrate the logical structure of an argument. A meta-analysis by Alvarez Ortiz (2007) showed that, at best, a one-semester university course using AM improved student critical thinking scores by approximately 0.6 Cohen's *d* compared to other courses. If *d* ≥ 1.0 could be achieved (e.g., in Phase 3), it would undoubtedly be of practical importance. At this stage of BARD's development (Phase 1), IARPA considered *d* ≥ 0.25 substantial enough to warrant further funding and development (Phase 2).

There is no natural scale for measuring reasoning performance, so the use of standardized effect size measures that are relative to observed variability is appropriate. But IARPA's blanket specification of Cohen's *d* as the standardized effect size measure was not optimal, and we pointed out some beneficial refinements. Cohen's *d* measures effect size in units of observed standard deviation (SD), and calculates this by pooling the SD of K and X. Better is Hedges' *g*, which also pools the SDs of K and X but corrects for a bias in Cohen's *d* where group sizes are small and unequal. For CREATE's purposes, better still is Glass' Δ, which uses only the SD of K. That's because, (i) “business as usual” is the relevant norm, and (ii) each new structured method is quite likely to have a different SD, and (iii) “business as usual” is therefore the only common standard of comparison for the four diverse methods. As Glass argued (Hedges and Olkin, 1985), if several treatments are compared to the control group, it's better just to use the control SD, so that effect sizes won't differ under equal means and different variances. Preserving the validity of the comparison in this way outweighs the slightly reduced accuracy of the estimation. An additional consideration is that for ANOVA-based analyses, it is usual to use a proportion of variance explained, e.g., by using partial eta-squared (η^2^) rather than Cohen's *d*. Accordingly, we report our effect sizes via two alternative measures below, but our preferred measure is Glass' Δ.

For the analysis of Problem A, an independent samples *t*-test was selected to assess the mean difference in rubric scores between K individuals and X teams, along with CIs for the two condition means. Effect size was reported using both Hedges' *g* and Glass' Δ.

For the analysis of the repeated measures data in Problems B and C, mixed-model ANOVA tests were selected to determine whether any difference in rubric scores is the result of the interaction between the “type of treatment” (i.e., membership of K or X) and “experience” (i.e., solving Part 1 or Part 2) alongside individual main effects for treatment and experience. Where the interaction term was not significant, rubric score differences between K and X were assessed through main effects for the type of treatment, and where the interaction term was significant, through the statistical significance of the simple main effects. Differences were computed using 80 and 95% two-sided Cousineau-Morey confidence intervals for condition means, and the 95% intervals were illustrated graphically. Effect size was reported using both partial η^2^ and Glass' Δ.

To explore potential marker bias due to report formatting, in each of the 5 problem-solving weeks we took three X and three K reports from participants and camouflaged them as reports from the opposing condition. We then randomly presented some blinded markers with the originals and others with the camouflaged versions. To analyze these 30 matched pairs of rubric scores, we used a mixed effects model with fixed effects (for condition and camouflage) and participant level random intercepts to test for any major bias.

## 5. Results

### 5.1. Attrition, Missing Values, and Bias

Attendance statistics for individual participants are shown in Table 2. To measure end-to-end attrition, the initial numbers are all participants who completed registration and confirmed their availability, and the final numbers are all participants who completed the task in Week 5. End-to-end attrition was about 50% in both conditions, although it was slightly lower (i.e., attendance was slightly better) in X than in K.

**Table 2:** Attendance by week: task completions and both week-on-week and end-to-end attrition, for K individuals, X individuals, all individuals, and X teams (along with the mean attendance per team).

**Condition**	**Attendance**	**Registration**	**Training**	**W1**	**W2**	**W3**	**W4**	**W5**	**Weekly**	**End-to-end**
K	Completed	58	51	44	34	31	28	28	–	–
K	Attrition %	–	12%	14%	23%	9%	10%	0%	11%	52%
X	Completed	198	140	130	122	112	114	105	–	–
X	Attrition %	–	29%	7%	6%	8%	−2%	8%	9%	47%
K+X	Completed	256	191	174	156	143	142	133	–	–
K+X	Attrition %	–	25%	9%	10%	8%	1%	6%	10%	48%
X Teams	Completed	25	25	25	23	23	22	21	–	–
X Teams	Attrition %	–	0%	0%	8%	0%	4%	5%	3%	16%
per X Team	Completed	–	5.6	5.2	5.3	4.9	5.2	5.0	5.2	–

Intermediate attendance numbers reveal that week-on-week attrition averaged about 10% in both conditions, although slightly lower in X than K, and tended to reduce as the experiment progressed. A notable difference between conditions is that in K the attrition during training was similar to subsequent problem-solving weeks, whereas in X the attrition during their more substantial training was much higher than K (more than double), but in subsequent weeks was almost always lower than K. Since all trained participants were allowed to resume participation even if they missed a week of problem-solving, it was possible for week-on-week attrition to be negative, which did occur when more X participants completed their task in Week 4 than Week 3.

In terms of teams, the number of X individuals available at each randomized allocation was sufficient to form 25 teams after registration, 23 before Problem B, and 22 before Problem C, with a mean size of 7.3 members for the experiment. Individual attrition resulted in a mean size of 5.2 members actively participating each week, which we expected would be sufficient for the BARD social process to confer significant benefits. Every team completed all of their weekly problem-solving tasks, except for one team in the final week, so 25, 23, and 21 X teams completed Problems A, B, and C, respectively. This equates to an end-to-end attrition for teams of only 16%, and a mean week-on-week attrition rate of only 3%. These are one third of the rates for X individuals, because the rest of the individual attrition occurred within teams^33^.

Since reports from Weeks 2 and 3 were analyzed collectively as part of the phased Problem B, only matched pairs were included: any report from either of these weeks was regarded as an incomplete datum and discarded if the K individual or X team did not also produce a report for the other week. Reports from Weeks 4 and 5 were treated similarly. Fortunately, in K individual attrition was noticeably reduced in the second week of a phased problem. This was not true for individuals in X, but as noted, only one such incomplete datum was produced by X teams. Missing data from X teams was also reduced via our automatic submission contingency: 20% of the X reports assessed were auto-submitted by the BARD system after the facilitator failed to submit, as shown in Table 3.

**Table 3:** X report submission method by week.

**Submitted by**	**W1**	**W2**	**W3**	**W4**	**W5**	**All**
Facilitator	25	16	15	18	17	91
Automation	0	7	8	4	4	23
Total	25	23	23	22	21	114

Attendance at the optional, compensated webinars was very good for K and excellent for X, as shown in Table 4. Relative to attendance the previous week for the associated upfront training or problem, webinar attendance was 73% in K and 92% in X.

**Table 4:** Individual attendance at optional feedback sessions.

**Condition**	**W1**	**W2**	**W4**	**All**
K	32	29	31	92
X	129	115	106	350
Total	161	144	137	442

Comparing the 30 camouflaged reports to their original counterparts, we did not detect any effect of the report format on the rubric scores awarded by markers [χ^2^(1) = 0.143, *p* = 0.706].

### 5.2. Test Assumptions

For each problem set, assumptions of normality and homogeneity of variances were assessed using Shapiro-Wilk and Levene tests respectively, applied across repeat-condition subgroups and assessed at 95% confidence.

The Shapiro-Wilk test rejected the null of normality only for K in Part 2 of Problem B (*p* < 0.001) and K in Part 1 of Problem C (*p* = 0.049), so these were further assessed using normal quantile-quantile (QQ) plots^34^. The QQ plot for K scores in Part 2 of Problem B was approximately normal, but revealed a single outlier individual performing well above the rest of K, and its temporary removal resulted in an acceptable Shapiro-Wilks outcome (*p* = 0.099). Anecdotal evidence from marked reports suggested that, contrary to the experimental guidelines, some of the highest performers in K used Bayesian analysis methods or tools (other than BARD) to produce their solutions. Such individuals will have increased the mean scores in K. However, we are averse to permanently removing this particular outlier and other unanticipated observations, especially given that these observations favor K. Also, it is well-known that ANOVA can tolerate data that is non-normal, and simulation studies using a variety of non-normal distributions have shown that the false positive rate is not affected substantially by violation of this assumption under an approximately normal distribution (Glass et al., 1972; Harwell et al., 1992; Lix et al., 1996). Visual inspections of QQ plots for K scores in Part 1 of Problem C indicate that, again, the distribution is sufficiently normal to allay concerns about inflated false positive rates.

Levene's test for homogeneity of variances was not significant for week–condition sub-groups in Problem A [*F*_(1, 69)_ = 1.319, *p* = 0.255] or Problem B [*F*_(3, 100)_ = 1.112, *p* = 0.348], but was significant for Problem C [*F*_(3, 84)_ = 5.406, *p* = 0.002]. Kim and Cribbie (2018) show that the impact of departures from homogeneity on false positive error rates are limited when sample sizes are close to equal. The sample sizes in Problem C were 23 for K and 21 for X, so we anticipate this departure from the homogeneity assumption will also have limited impact on false positive rates in final outcomes.

### 5.3. Analysis

**Figure 3:**
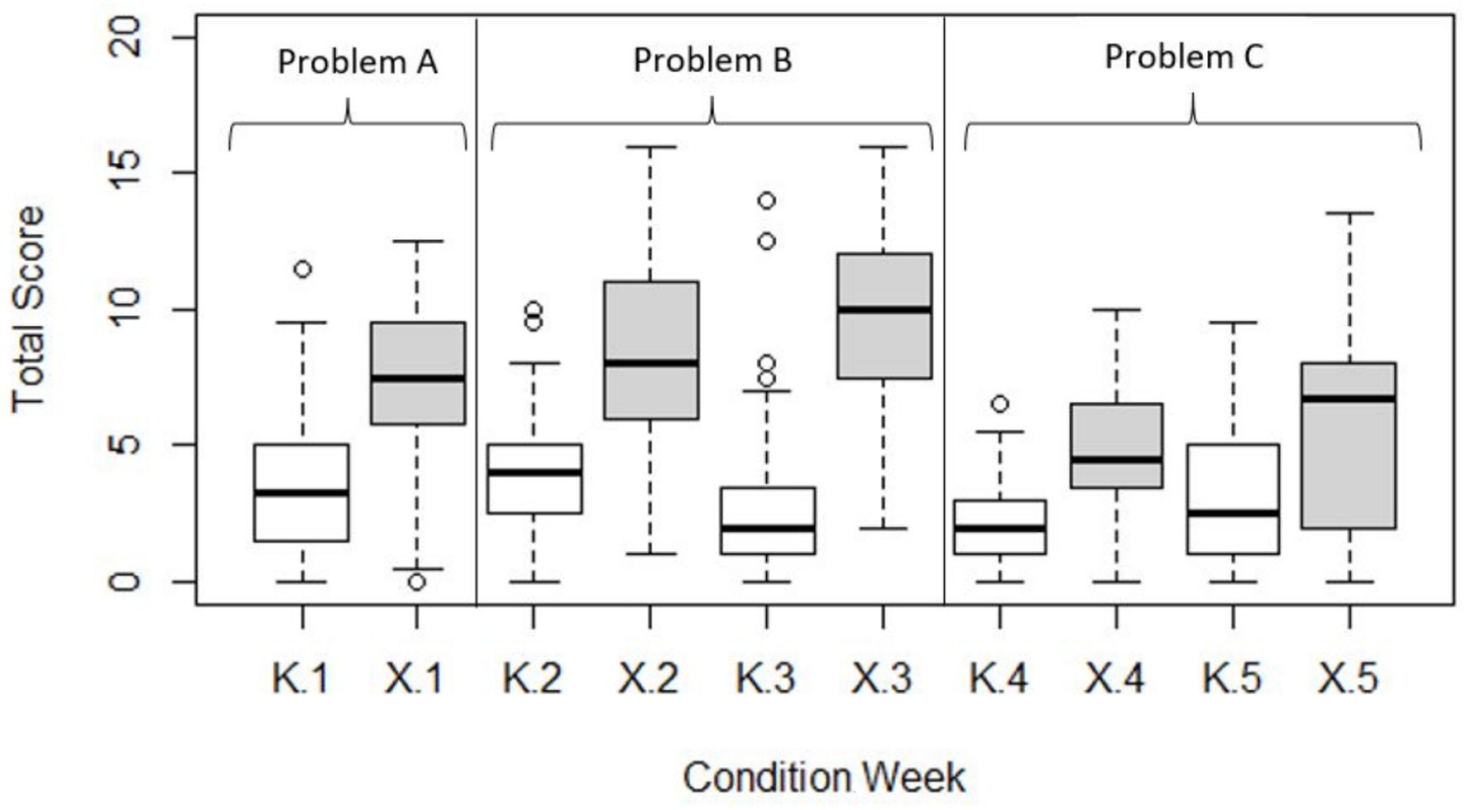
Boxplot of score quartiles for each condition in each problem-solving week. Medians are represented by the thicker horizontal bars. Outliers are represented by circles, and defined as further than 1.5 times the interquartile range from their condition median.

Figure 3 is a set of box plots summarizing and exploring the quartile distribution of rubric scores, after balancing data for attrition by dropping observation pairs with missing values. Median scores indicate that the “middle” team in X always outperformed the “middle” individual in K in all weeks, providing some initial support for our main hypothesis. While the higher quartiles of K predominantly overlap with the lower quartiles of X, as noted, there are a few outliers in K that perform as well as their high performing X counterparts. Median scores in K vary little across weeks. The superiority of the medians in X is most striking for Problems A and B, and somewhat less for Problem C.

#### 5.3.1. Problem A: Smoking and Cancer

**Figure 4:**
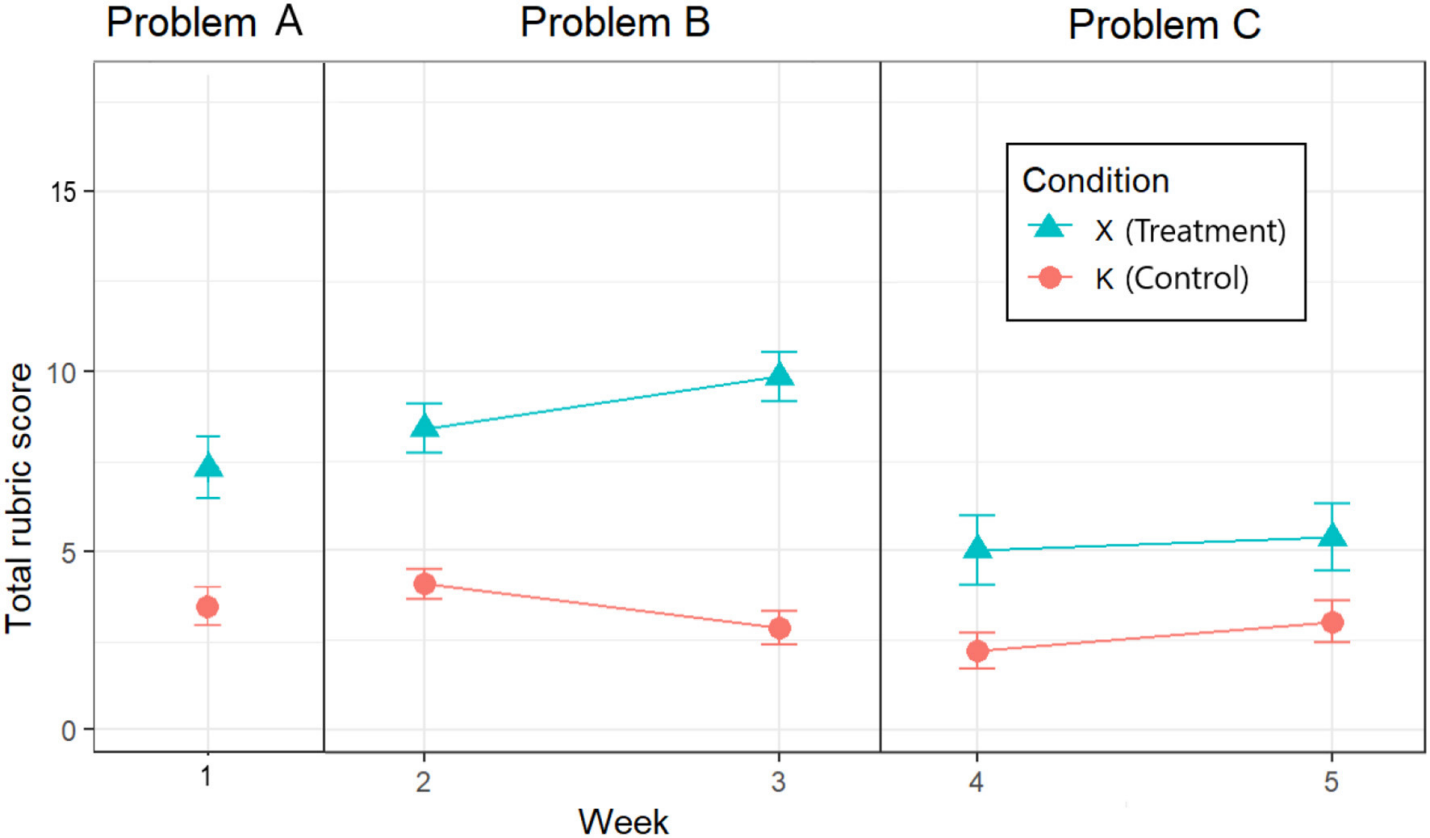
Mean scores with their 95% CIs for each condition in each problem-solving week.

For Problem A, the difference in mean scores between X and K was statistically significant [*t*_(91.223)_ = 7.799, *p* < 0.001] using an independent samples (Welsch) *t*-test. 80% and 95% confidence intervals were calculated around each condition's mean score (see Table 5 and Figure 4) and do not overlap, further indicating significantly higher mean scores in favor of BARD.

**Table 5:** Mean scores with their 80% and 95% CIs for each condition in Problem A.

**Cond**.	**Week**	**N**	**Mean**	**SD**	**SE**	**95% CI**	**80% CI**	**Max**
K	1	44	3.545	2.283	0.344	[2.851–4.240]	[3.097–3.993]	13
X	1	25	7.370	2.775	0.555	[6.225–8.516]	[6.639–8.101]	13

Given the unequal sample size (K = 44, X = 25), we computed the adjusted Hedges' *g* effect size of 1.44, while Glass' Δ = 1.6. On either measure, this is considered a very large effect.

#### 5.3.2. Problem B: Kernel Error

For Problem B, inspecting the Week × Condition mean rubric scores for Weeks 2 and 3 depicted in Figure 4, we can see that the difference between the control and experimental conditions increases, which suggests a Week × Condition interaction. Indeed, our 2 × 2 mixed ANOVA showed a statistically significant interaction between experimental condition and problem week [*F*_(1, 50)_ = 8.93, *p* < 0.001]. The main effect of experimental condition was significant [*F*_(1, 50)_ = 86.46, *p* < 0.05], while the mean effect of exposure week was not [*F*_(1, 50)_ = 0.06, *p* = 0.81].

Adjusted confidence intervals as described by Morey (2008) were calculated around each Week × Condition mean score (see Table 6 and Figure 4), and do not overlap for K and X in either Week 2 or Week 3, further indicating significantly higher mean scores in favor of BARD.

**Table 6:** Mean scores with their 80% and 95% CIs for each condition in Problem B.

**Cond**.	**Week**	**N**	**Mean**	**SD**	**SE**	**95% CI**	**80% CI**	**Max**
K	2	29	4.069	2.437	0.320	[3.428–4.710]	[3.654–4.484]	18
X	2	23	8.402	3.575	0.527	[7.341–9.464]	[7.717–9.088]	18
K	3	29	2.845	2.721	0.357	[2.129–3.560]	[2.382–3.308]	16
X	3	23	9.848	3.591	0.529	[8.781–10.914]	[9.159–10.536]	16

The size of the main effect of condition as measured by the generalized η^2^ is 0.53, which is considered very large (Bakeman, 2005), while Glass' Δ = 2.2, which is considered huge. The generalized η^2^ effect size for the Week × Condition interaction, 0.06, is considered small^35^.

#### 5.3.3. Problem C: Cyberattack

For Problem C, inspecting the Week × Condition mean rubric scores for Weeks 4 and 5 depicted in Figure 4, we can see that the difference between the control and experimental conditions is similar, which suggests no Week × Condition interaction. Indeed, our 2 × 2 mixed ANOVA showed there was no statistically significant interaction between experimental condition and problem week [*F*_(1, 42)_ = 0.35, *p* < 0.56]. The main effect of experimental condition was significant [*F*_(1, 42)_ = 17.68, *p* < 0.05], while the main effect of exposure week was not [*F*_(1, 42)_ = 2.58, *p* = 0.12].

Again, adjusted confidence intervals were calculated around each Week × Condition mean score (see Table 7 and Figure 4), and do not overlap for K and X in either Week 4 or Week 5, further indicating significantly higher mean scores in favor of BARD.

**Table 7:** Mean scores with their 80% and 95% CIs for each condition in Problem C.

**Cond**.	**Week**	**N**	**Mean**	**SD**	**SE**	**95% CI**	**80% CI**	**Max**
K	4	23	2.208	1.751	0.253	[1.700–2.717]	[1.880–2.537]	22
X	4	21	5.012	3.070	0.474	[4.055–5.968]	[4.395–5.629]	22
K	5	23	3.022	1.972	0.291	[2.436–3.607]	[2.643–3.400]	16
X	5	21	5.369	3.009	0.464	[4.431–6.307]	[4.764–5.974]	16

The size of the main effect of condition as measured by the generalized η^2^ is 0.24, which is considered large (Bakeman, 2005), while Glass' Δ = 1.4, which is considered very large.

## 6. Discussion

### 6.1. Likely Causes and Effects of Attrition

For Delphi studies, which necessarily require participants to respond for two or more rounds on the same test problem, attrition rates per round can be high, accumulate to extremely high levels, and threaten to bias the results (Toma and Picioreanu, 2016). Some typical *initial* attrition rates (i.e., at the second round) reported in the literature are approximately 15% (Elwyn et al., 2006), 30% (Bradley and Stewart, 2002), and 50% (Moreno-Casbas et al., 2001; Goluchowicz and Blind, 2011). In comparison, the 10% weekly attrition rates we achieved were very low, and our end-to-end rate of 50% after six rounds was, although high, about equal to the attrition rate seen after one round in the latter studies and our piloting. Consequently, unlike the larger IARPA study, we managed to cater for and reduce individual attrition sufficiently to obtain statistically significant results, assisted by our participant compensation, social engagement, team sizes, and auto-submission.

In the aborted IARPA study, in which four contrasting systems were being tested along with a control condition similar to our own, the length of upfront training varied between systems, and attrition rates during this training were roughly proportional to its duration. Since upfront training was at least twice as long in X as in K, the doubled attrition in X compared to K is consistent with the IARPA study, and does not imply that our training was particularly difficult.

In the problem-solving weeks, two possible causes for the slightly lower individual attrition in X compared to K are benefits of working as a group: the mean task burden per individual can be reduced by distributing it (often not evenly!) amongst team members, and the social interactions involved in the task can make it more attractive. Another possible cause is a benefit of using BNs: the tool may have seemed better suited to the task, encouraging participants to persist with it.

We expect the main introduced bias due to attrition was that participants who felt more competent at the task were more likely to show up for subsequent rounds, potentially improving average performance in the condition. Performance in both K and X may have progressively benefited from this, but the principal concern here is that they may not have benefited equally, thus contributing to our effect sizes one way or the other. Both K and X involved using techniques (mathematical and modeling respectively) that some individuals would have been able to use better than others, so in this respect it isn't clear which condition's individuals would have benefited more. There are, however, two social factors that clearly should have reduced the benefit to X teams. First, participants who felt less competent should already have had less impact than their team members on the team report, so their absence probably didn't improve the team responses as much per report as attrition in K. Second, in a group social process like Delphi, less capable participants may still make a positive net contribution to a group report, so it is possible that their absence actually made X reports worse. Finally, although it may have been obscured by the variation in problem-solving tasks, there was no observable trend of increasing effect sizes over the 5 weeks as the level of attrition increased. For all these reasons, it seems very unlikely that attrition made a major positive contribution to our headline result: that X consistently outperformed K.

There is one other, important reason why IARPA, at least, was sanguine about possible attrition bias. The intended use of any CREATE system was not to make it a compulsory tool, replacing business as usual for all analysts. Rather, it was to make it available as an optional alternative for any analysts who are attracted to it and voluntarily persist with it. This was, similarly, expected to select those who feel more competent using the system, and create a self-selection and attrition bias far greater than any in our experiment. Hence, although we strived to minimize attrition, any attrition bias there may have been in our experiment will only have made our results a more accurate indicator of likely performance for IARPA's intended use.

### 6.2. Effects of Problem Difficulty

We know that much more complex problems of a similar kind can be solved accurately by BN experts using the tools in X, and aren't tractable for anyone using the tools in K. So, we expected that the increase in complexity in the second phase of Problems B and C would translate into a bigger advantage for X over K. However, the advantage detected in B was small, and not detected at all in C. It may be that the increases in complexity and/or the ability of our participants to use BN models to overcome it was not as great as we supposed.

Since the second phase of Problem B involves the “explaining away” cognitive difficulty, whereas the second phase of Problem C involves the “dependent evidence” cognitive difficulty, this may suggest that explaining away is more difficult to understand than dependent evidence. However, this interpretation would be unwarranted. We used only one example of each difficulty, so there are numerous confounds; and this effect-size ordering was not observed in the SoloBARD experiment. Measuring the relative difficulty of various cognitive difficulties would require many further, more careful comparisons.

### 6.3. Robustness and Size of Effect

**Figure 5:**
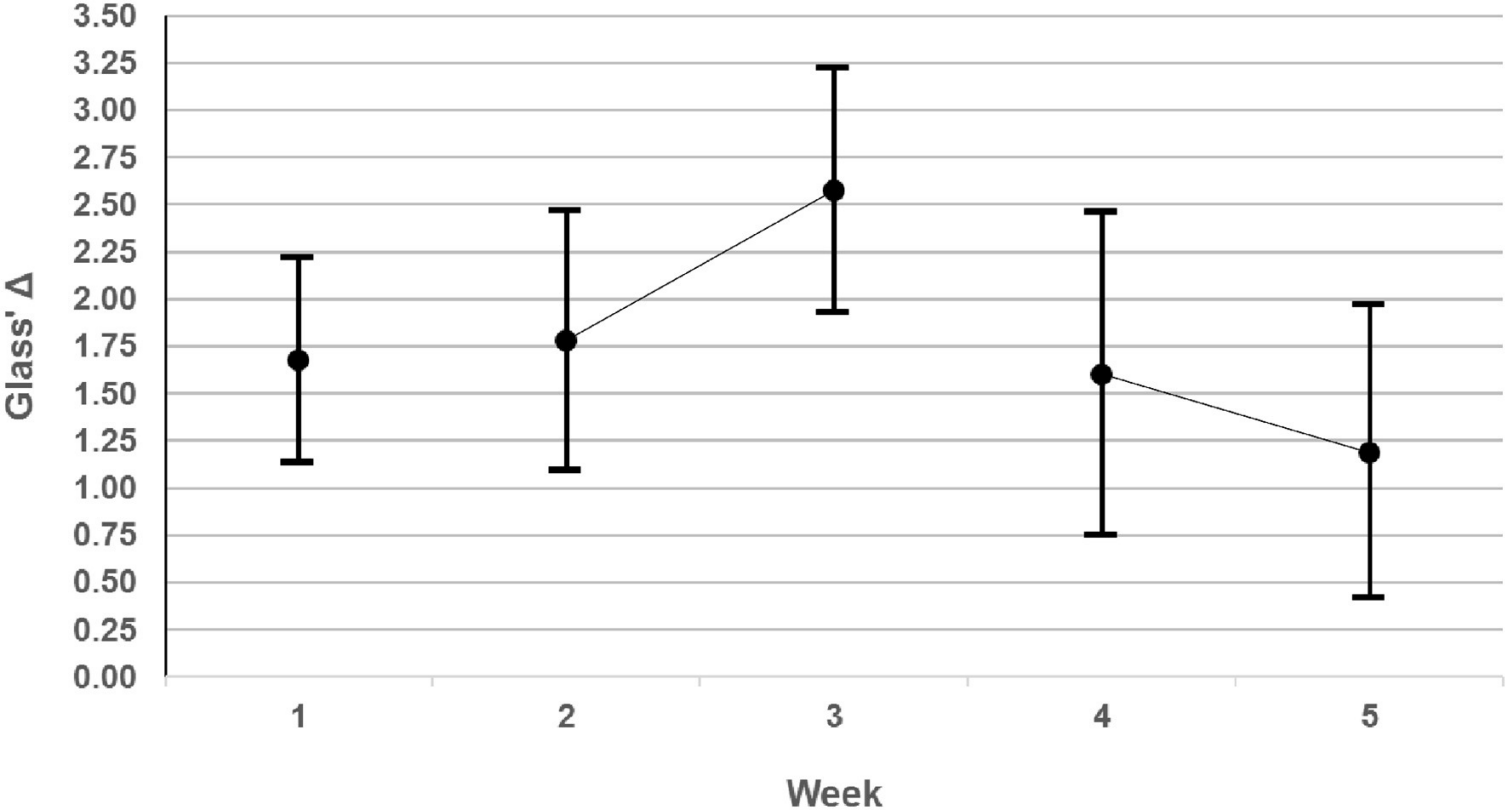
Effect sizes measured by Glass' Δ with their 95% CIs for each week.

The superior performance of X over K was a robust effect across our three problems, since it was confirmed independently for each. On our preferred measure, the effect sizes were all very large to huge (Glass' Δ 1.4–2.2), and their 95% CIs are shown graphically by week in Figure 5. On any standard measure, they greatly exceeded CREATE's initial target of a small effect size, and indeed, achieved in Phase 1 for simple problems the large effect size desired in Phase 3 for more complex Problems.

It is interesting to compare the performance of our participants to those in the SoloBARD experiment, which used a similar set of three problems (a different problem instead of our Problem A, but exactly the same Problems B and C). Unexpectedly, SoloBARD participants performed better than ours. However, this was mainly in the control condition—so, as expected, our BARD users beat our controls by a greater margin than the SoloBARD users beat their controls. Specifically, SoloBARD control individuals performed much better (obtaining 32% of the available points) than our control individuals (obtaining only 17%), while SoloBARD experimental individuals performed only slightly better (obtaining 48%) than our BARD experimental teams (obtaining 41%). Consequently, BARD achieved double the mean effect size (Glass' Δ = 1.7) of SoloBARD (Glass' Δ = 0.8).

There were multiple differences between the two experiments that may have affected performances, so we must be cautious in attributing specific causes to the differences in results. However, we see no factor likely to have benefited *only* the SoloBARD controls compared to our K. On the contrary, while in both experiments control individuals received IARPA's Guide to Good Reasoning, our K individuals also received some training in pen-and-paper probability calculation techniques, which should have improved their relative performance—yet this effect is not evident, perhaps because it is swamped by other factors. In contrast, there are several plausible causes for better performance in *both* the control and experimental conditions of SoloBARD compared to our K and X: (i) superior ability of participants, who were drawn solely from the University College London experimental participant pool rather than recruited on the more *ad-hoc* basis described in section 4, (ii) in-lab testing rather than online, which tends to improve motivation and compliance, and (iii) offering substantial and extensive financial bonuses for good performance (to supplement a modest hourly rate), rather than just offering a generous hourly rate. It is possible that these factors made more difference to the relative performance of the control conditions than to the experimental conditions. However, there is a more obvious explanation for the greater outperformance of the experimental over the control condition in our BARD experiment: our X participants benefited from working in small groups. This is consistent with the general prior literature on Delphi and our specific prior experiment with Delphi in BARD, as summarized in sections 2.4, 2.5, and 2.7.

In summary, there were clearly significant factors driving down performance in our experiment compared to the SoloBARD experiment, and there may have been an interaction effect that contributed one way or the other to our effect sizes. Nevertheless, with that caveat, the doubled effect size achieved by BARD in comparison to SoloBARD suggests, and provides some cumulative evidence, that our social processes make a substantial positive contribution in addition to the substantial positive contribution made by BN construction.

### 6.4. Quality of Reports, Causal Models, and Training

As expected, the mean proportions of the available rubric points obtained by participants were low, even when assisted by BARD. As discussed in section 4.2, participants are unlikely to provide a high proportion of the specific items the rubric rewards. Our rudimentary AI tool was designed to suggest possible text to include in justifications, but at this early stage of development it was not able to suggest all the relevant points, and apparently it had limited effect.

Given that our X participants were supposed to achieve better written reports than K by constructing BN models, it is natural to ask how accurate their models turned out to be, and how well-correlated this was to the quality of their written reports. In Bolger et al. (2020), our BARD team members assessed the quality of BN structures by measuring the difference between these and normatively correct “gold-standard” structures using “edit distance,” which is the most well-known structural measure in the literature (e.g., Spirtes et al., 2000). However, this approach was facilitated by requiring participants to choose variables out of a set provided, and not requiring probabilities to be entered in the models, thus avoiding both sources of variation in participant answers. Furthermore, the aim was to compare the relative quality of structures produced by individuals before and after peer feedback, not their absolute quality. Here, even if we used a broader measure of the overall quality of the BNs produced by our X teams, there would be no meaningful comparison to evaluate how well our X teams performed. The only way to compare X to K performance is to measure the quality of their written reports, which they both produce, and are designed to implicitly test the accuracy of the BNs constructed by X teams via the accuracy of their answers. The same inherent limitation applied to the SoloBARD experiment. However, we will make all the BNs produced by our X teams available for subsequent research.

For similar reasons, it is difficult to assess the skill level in BN construction achieved by our X teams. There is, as yet, no standard test for BN-modeling difficulty or ability, so we can't quantify more precisely the difficulty of building our problem BNs or the ability achieved through our minimal training. However, it is notable that, as in our two previous experiments, our BARD users received very little BN training by industry standards, and yet they were able to construct BNs well enough to outperform control participants on our probabilistic reasoning problems. This provides some welcome evidence that intelligence analysts, for example, can be quickly trained to use BNs using our online resources. We are also confident that with further training and experience, X teams would substantially improve their BN building skills and consequently their written reports.

## 7. Conclusion

Our results show that BARD is an extremely promising tool for intelligence analysis that warrants further research. Compared to business as usual, it already performs much better on simple test problems. Compared to existing BN software, it offers a unique integration of BN construction with a Delphi-style collaborative workflow, high-quality online training and help, and a structured template for written reports with complementary text explanations automatically generated from the BN. Furthermore, there is enormous potential for further research and improvement: in developing more complex problems, in developing BARD's features, and in testing their individual and combined efficacy on those problems. There are also numerous potential applications for BARD outside intelligence analysis, since many areas—including those to which BNs have already been introduced—require reasoning and decision making under uncertainty.

More generally, our results provide some cumulative evidence (in addition to prior theory and experiments) for the utility of BARD's key components:

- Good online training allows people who are not BN experts to construct BNs, minimizing the need for a facilitator who is a BN expert.
- Where time permits, BN construction can be used effectively for probabilistic reasoning problems. This helps to avoid numerous types of causal and probabilistic reasoning difficulties, and adds precision.
- Small group collaboration, via RT Delphi in particular, can be used successfully for BN construction. This allows multiple viewpoints to be debated and combined to produce a better result.

Three issues for further research deserve particular emphasis:

1. We must test the efficacy of probability estimation. Our team showed that it is possible and necessary to develop a new type of test problem for probabilistic reasoning: sufficiently challenging, yet simple enough to assess (with many normatively correct elements in the solution). More complex problems of this sort must be developed that include the estimation of probabilities by experiment participants, rather than relying entirely on precise parameters specified in the problem statement. BARD's built-in capacities for eliciting and combining probability estimations can then be rigorously tested.
2. Our social processes, in addition to RT Delphi, include components such as discussion boards and the rating of other team members' work. These components can be evaluated and optimized individually and in combination. If such components work sufficiently well, then in many applications BARD could dispense with the human facilitator altogether without much loss.
3. Our automated verbal explanations were novel and promising, but we have not yet measured their contribution. Moreover, we now believe this XAI tool would be better implemented as a combination of visual and verbal features that are more interactive. Our spin-off project, mentioned in section 3.4, will investigate this in detail^36^.

## Data Availability Statement

The datasets generated for this study are available on OSF or on request to the corresponding author.

## Ethics Statement

The studies involving human participants were reviewed and approved by Human Research Ethics Committee, Monash University. The patients/participants provided their written informed consent to participate in this study.

## Author Contributions

All of the authors were involved in the design, construction and testing of the BARD application to greater or lesser extents, excepting MO and YL. The latter two as well as AO were involved in data analysis, with MO (from Statistics, Monash) doing much of the heavy lifting. RP was involved in the organization and day-to-day oversight of the experiments, while ST and AO organized participants, ran the BARD webinars and were online during the synchronous BARD sessions for technical support as required. KK and EN conducted the control group webinars. KK led the design and oversaw the running of the experiment, as well as leading the BARD project as a whole and wrote much of this paper. AO drafted the manuscript. AN made significant rewrites. In response to referee feedback, EN managed supplementary data analysis and reporting, and made extensive revisions and additions to the manuscript. All authors reviewed drafts, providing feedback, and suggesting edits.

## Conflict of Interest

The authors declare that the research was conducted in the absence of any commercial or financial relationships that could be construed as a potential conflict of interest.

## Footnotes

^1^IARPA is an organization within the Office of the Director of National Intelligence responsible for leading research to overcome difficult challenges relevant to the United States Intelligence Community. For more details see https://www.iarpa.gov/.

^2^CREATE webpage: https://www.iarpa.gov/index.php/research-programs/create.

^3^BARD webpage: http://bard.monash.edu/.

^4^To view and interact with BARD (including solved demonstration problems), to view the BARD training e-course, or to use BARD for academic research purposes (including refereeing and replications), please email Prof. Ann Nicholson at ann.nicholson@monash.edu.

^5^Hugin website: https://www.hugin.com/.

^6^GeNie website: https://www.bayesfusion.com/.

^7^Netica website: https://www.norsys.com/index.html.

^8^Agena Risk website: https://www.agenarisk.com/.

^9^BayesiaLab website: http://www.bayesia.com/.

^10^Elvira website: http://leo.ugr.es/elvira/.

^11^R BN website: http://www.bnlearn.com/.

^12^BNT website: https://github.com/bayesnet/bnt.

^13^SamIam website: http://reasoning.cs.ucla.edu/samiam/.

^14^BayesPy website: https://pypi.org/project/bayespy/.

^15^BayesiaLab website: http://www.bayesia.com/events.

^16^Bayesian Intelligence website: https://bayesian-intelligence.com/training/.

^17^For a glimpse of this upfront training, see “BARD Screenshots” at https://tinyurl.com/bard-publications.

^18^This “explainable AI” (XAI) feature is now undergoing further development as part of a spinoff project involving several BARD researchers. “Improving human reasoning with causal Bayes networks: a multimodal approach”is a major 3-year project at Monash University and the University of London funded by the Australian Research Council. See https://dataportal.arc.gov.au/NCGP/Web/Grant/Grant/DP200100040.

^19^“Experiment Design” available at: https://bit.ly/2OBVBCc.

^20^The Open Science Framework is an open source software project that facilitates open collaboration in science research. See for more information: https://osf.io/.

^21^“Short Quiz” available at: https://bit.ly/2K6Nj6N.

^22^GiftPay website: https://www.giftpay.com/.

^23^“Smoking and Cancer - Problem Statement” available at: https://bit.ly/2V2rwl4.

^24^“Smoking and Cancer - Rubric” available at: https://bit.ly/2v92emU.

^25^“Training Presentation - Group X” available at: https://bit.ly/2VnjW1d.

^26^Admittedly, there was no active monitoring or intervention in this experiment to prevent facilitators from flouting this training by industriously building their own independent BN solution and writing a report based solely on it—but we received no complaints from analysts that such dictatorial behavior occurred.

^27^For the IARPA experiment, we defined a similar set of rules to classify and submit a report as “non-deficient,” and slightly stricter rules requiring participation from several analysts (per the intended social process) to classify it as “Ready-to-Rate.” The latter would have been a better basis for assessment if the sample sizes had been sufficient. “BARD Report Flags” available at: https://bit.ly/2CTk3u7.

^28^“Control Group Plan” available at: https://bit.ly/2YOkEXo.

^29^“Guide to Good Reasoning” available at: https://bit.ly/2WJtpQJ.

^30^“Training Presentation - Group K” available at: https://bit.ly/2IduCMo.

^31^“Assessment Guide” available at: https://bit.ly/2G1YUjD.

^32^“Experiment Design” available at: https://bit.ly/2OBVBCc.

^33^“Attrition Rates” available at: https://bit.ly/2K7wFnG.

^34^“Quantile Quantile Plots” available at: https://bit.ly/2YMHYot.

^35^“ANOVA Tables and Main Effects” available at: https://bit.ly/2YPRDec.

^36^“Improving human reasoning with causal Bayes networks: a multimodal approach.” See https://dataportal.arc.gov.au/NCGP/Web/Grant/Grant/DP200100040.
